# How Psychological Symptoms Mediate Perceived COVID-19 Stress With Identity Distress in Emerging Adults

**DOI:** 10.1177/21676968231185171

**Published:** 2023-06-22

**Authors:** Barbara M. Gfellner, Ana I. Cordoba

**Affiliations:** 1 Department of Psychology, 1916 Brandon University, Brandon, MB, Canada; 2 Department of Developmental and Educational Psychology, 311343 University of Valencia, Valencia, Spain

**Keywords:** COVID-19 stress, psychological symptoms, identity distress, CCAPS-34, emerging adults

## Abstract

The global pandemic has been associated with substantial elevation in mental health problems among emerging adults. In this study, we examined psychological symptoms in relation to perceived COVID-19 stress and disturbances with identity development (identity distress) among university students in Canada and in Spain during the second wave of the pandemic. Spanish students indicated greater identity distress than their Canadian counterpart, and they reported higher perceived COVID-19 stress. The predicted associations were supported among perceived COVID-19 stress, psychological symptoms, and identity distress for both groups, and psychological symptoms mediated the linkage between perceived COVID-19 stress and identity distress. These results underscore the enduring influence of psychological symptoms in relation to perceived COVID-19 stressors and identity development with implications for other serious contextual events and suggestions for student support and clinical intervention.

## Introduction

The novel coronavirus (COVID-19) was declared a worldwide pandemic on March 11, 2020 (WHO, 2020). It has been responsible for unprecedented deaths, financial instability, excessive physical and mental health problems, and societal disruption globally (Cavicchioli et al., 2021; McGinty et al., 2020; Pierce et al., 2020; Wirkner et al., 2021); and it continues to exert a disruptive effect on individuals’ functional well-being (Barone et al., 2023; Daly & Robinson, 2022; Taylor & Asmundson, 2020). Across the lifespan, emerging adults (EA), defined as young people between 18 and 29 years of age (Arnett et al., 2014), have been found to experience the greatest negative impact of COVID-19 with particular concerns among university students (Aknin et al., 2022; Araujo et al., 2020; Kujawa et al., 2020; Lederer et al., 2021; Muller et al., 2023; Tasso et al., 2021).

Early in the pandemic before the availability of vaccines, universities including residences were closed and remote, online, or hybrid learning was instituted. Students’ social interaction was confined to their household with many returning to live with parents. Health worries for oneself, others, and general uncertainty for the future were prominent. The pervasive global lockdowns created extraordinary developmental disruptions relative to prior cohorts that led to immediate and long-term challenges for many students as seen in an exacerbation of the psychological difficulties that often accompany this phase of development (Gruber et al., 2021; Tasso et al., 2021).

Apart from mental health and psychological symptoms, there has been less research emphasis on identity development during the pandemic. This is surprising as identity development is a prominent psychosocial task of EA when young people search, experiment, and evaluate options in the process of establishing a coherent sense of self that includes consideration of the future (Arnett, 2016). Constructing one’s identity typically involves challenges and uncertainty, yet this process may be extremely disruptive and distressing for some individuals (Waterman, 1988; Wiley & Berman, 2013). Difficulties with identity issues and psychological problems have been accentuated among people in the context of deleterious events such as economic recessions, natural disasters, political disruption, and wars (Berman, 2016; Berman et al., 2020; Gfellner et al., 2019; Gfellner & Cordoba, 2020b; Parker et al., 2016; Scott et al., 2014; Wiley et al., 2011). Indeed, the contextual complexities of the COVID-19 pandemic would be expected to interfere with identity development as reflected in identity problems and distress.

The present study draws on the sixth wave of a repeated cross-sectional project on adaptation to university among students in Spain and Canada. Earlier pre-pandemic findings indicated higher identity distress as well as superior ego functioning and adjustment at university among Spanish students in comparison with Canadian cohorts (Gfellner & Cordoba, 2017; 2020b). Further study showed that students in Canada evidenced a normative identity development progression relative to those in Spain and Spanish students maintained elevated identity distress along with advanced identity resolution (Gfellner & Cordoba, 2020a). In these studies, Spain and Canada were proxies for different contextual situations (high youth unemployment versus a stable economy and distal versus proximal disruptive conditions). The findings reflected a resilience among Spanish students in the face of contextual disturbance. Indeed, resilient people often demonstrate hardiness when faced with adverse events and environments (Masten, 2021). In the current study COVID-19 provided the context of an unprecedented global health pandemic in which to investigate macro-contextual influences as well as possible cultural differences in the pervasiveness of identity difficulties and associations with psychological symptoms and perceived COVID-19 stress.

This study examined the prevalence of psychological symptoms and identity distress among students in Spain and Canada and how these factors are associated with the students’ perceived COVID-19 stress. As part of a repeated cross-sectional study baseline data was available to determine the extent of change among students in these countries during the pandemic. The findings are expected to complement extant research on the mental health of EA during the pandemic, to provide information on the extent to which concomitant identity development may be impeded, and to unpack associations between psychological symptoms, identity distress, and students’ perceptions of COVID-19 stress. It is anticipated that the results will contribute to our understanding of psychosocial development during EA and offer directions for intervention with continuing ramifications of the pandemic and subsequent macro-contextual catastrophes.

### Mental Health and Psychological Symptoms

Numerous studies have documented the impact of COVID-19 in terms of an increased incidence of mental health problems, notably, anxiety, depression, and stress (Barone et al., 2023; Barendse et al., 2023; Cavicchioli et al., 2021; Hoyt et al., 2021; Pan et al., 2021). This has been indicated globally and with young people the most vulnerable demographic (Aknin et al., 2022; Daniali et al., 2023; Muller et al., 2023; Robinson et al., 2022; Varma et al., 2021). This is alarming since psychological difficulties tend to become evident initially during this age span (Persike et al., 2020; Tanner, 2015). Indeed, those who reported the greatest COVID-19 stress indicated the most psychological symptoms (Holmes et al., 2020; Howard et al., 2022; Kujawa et al., 2020). These findings are consistent with the increased incidence of mental health problems such as anxiety, depression, post-traumatic stress disorder, substance abuse, and domestic violence among individuals who experienced natural disasters, war, terrorist attacks, and societal disruptions (Galea et al., 2020).

At the same time Muller et al. (2023) reported higher levels of resilience using ecologically momentary assessments of mood and depression in the early phases of COVID-19 among adults in the United States, United Kingdom, and Germany. According to these authors, the nature of the adverse events may elicit different coping responses that affect functional well-being. Natural disasters such as hurricanes and earthquakes, as well as terrorist attacks are time-bound while in comparison the duration of the pandemic is uncertain with an impact that is constantly changing over time. In an extensive review Manchia et al. (2022) indicated resilience as individuals may learn to adapt to changing circumstances as the pandemic extends over time. Haikalis et al. (2022) found decreases in anxiety and depression among college students who reported positive advantages associated with the pandemic. To date, most published studies have focused on the first year of the pandemic (Aknin et al., 2022). More recently, Daly and Robinson (2022) reported an increase in psychological distress with subsequent lockdown during the second wave of the pandemic in the UK. Indeed, continued evaluation of mental health indicators over subsequent waves is essential to elucidate the factors associated with resilience in the face of such macro-environmental disasters.

### Identity Development and Identity Distress

According to lifespan theory identity development is the major psychosocial task for young people in the transition to adulthood (Erikson, 1968). It involves searching, exploring, querying, and evaluating potential identity alternatives and making commitments in the process of constructing a coherent self-conceptualization that integrates the past with the present to subsequently influence future decisions (Erikson, 1968; Marcia, 1966; Mitchell et al., 2021; Schwartz et al., 2013). The university environment is an optimal context for identity development as it provides students with an extended time to explore and actively question aspects of their lives including education, vocational pursuits, relationships, values, and lifestyle choices before they have fully assumed adult roles and responsibilities (Arnett, 2016). Although distress with identity issues is expected in the process of negotiating one’s sense of self, some individuals become overwhelmed and identity distress interferes seriously with their daily functioning (Berman et al., 2004; Wiley & Berman, 2013). Based on the criteria in the DSM nomenclature of identity dysfunction, Berman et al. (2004) constructed the Identity Distress Scale (IDS) to measure excessive and prolonged uncertainty over several identity-related issues: long-term goals, career choice, friendships, sexual orientation and behavior, religion, moral values, and group loyalties with consequent distress and disruption to normal functioning. Identity distress is associated positively with moratorium, the identity status that involves actively exploring identity issues before making any commitments (Berman et al., 2004), with maladaptive identity exploration and inversely with identity consolidation (Gfellner & Cordoba, 2020a; Palmeroni et al., 2019; Sica et al., 2014).

Developmental psychopathology emphasizes the role of identity distress in mental health difficulties (Kaufman et al., 2014). As expected, elevated identity distress is seen in clinical samples (Wiley & Berman, 2013), high-risk adolescents (Hernandez et al., 2006), and college students who were diagnosed and/or received treatment (Samuolis et al., 2015). In terms of mental health and wellbeing, identity distress has been associated with internalizing and externalizing symptoms (Hernandez et al., 2006; Papazova et al., 2018; Wiley & Berman, 2013), negative affect (Samuolis & Griffin, 2014), posttraumatic stress (Scott et al., 2014; Wiley et al., 2011), negative body image (Kamps & Berman, 2011), psychosocial immaturity, and maladjustment at university (Gfellner & Cordoba, 2017). Gfellner and Cordoba (2020b) found that psychological symptoms mediated between identity distress and social, academic, and personal-emotional adjustment at university among Spanish and Canadian students, and country/context and psychological symptoms were independent predictors of identity distress. Taken together, the findings suggest that identity distress is a common problem for many young people and especially for those with mental illness.

As indicated previously, EA is a period when psychological problems tend to become evident for the first time with notable escalation among post-secondary students (American College Health Association (ACHA), 2019; Auerbach et al., 2018; Centre for Collegiate Mental Health (CCMH), 2015; Persike et al., 2020; Tanner, 2015). Indeed, this increase in mental health difficulties has been found to complicate and interfere with identity synthesis and integration (Berman & Montgomery, 2014; Klimstra & Denissen, 2017; Palmeroni et al., 2019).

### Identity Distress and Context

From the bioecological perspective (Bronfenbrenner, 2005) macro-environmental factors exert important influences on behavior and psychological well-being. This may be reflected in the predicted rise in identity distress and identity problems due to globalization, increased immigration (Berman & Weems, 2012) and other contextual factors including social and political changes, wars, economic recessions, and natural disasters (Scott et al., 2014; Wiley et al., 2011). Such events increase the challenges in identity issues for young people in the transition to adulthood with concomitant increase in identity distress and delayed identity development. As noted above, the COVID-19 pandemic has been associated with a dramatic increase in mental health problems among university students throughout the world (Aknin et al., 2022; Manchia et al., 2022; Muller et al., 2023). The pervasive global lockdowns and concurrent heterogenous factors that permeate all socioecological levels (Ettekal & Agans, 2020; Gruber et al., 2021; Salmon, 2021; Velez et al., 2022), coupled with the ongoing uncertainty of the pandemic may obfuscate identity development and for many EAs this would be expected to be evident in a rise of identity distress.

### Pre-Existing Mental Health Problems

From the clinical perspective using Global Burden of Disease data for 2020 from 204 countries, Lokman and Bokting (2022) outlined a conceptual model to underscore the recurrent self-reinforcing role of mental health difficulties (anxiety and depression) during and after the COVID-19 pandemic. Given that anxiety and depression are comorbid, meaning they can trigger each other and are highly recurrent, these disorders are considered risk factors for subsequent episodes leading to more mental health problems. In a prospective 7-year follow-up from early adolescence to emerging adulthood, Crocetti et al. (2013) found that untreated externalizing symptoms among youth led to further delays in identity development and this reinforces an elevation in externalizing behavior over time. As well, EA who developed an internalizing disorder or showed an increase in depression and anxiety during this period have an increased risk of economic hardship and dependence on parents or the welfare system (Melkevik et al., 2016).

Other studies indicated that those psychologically vulnerable before the pandemic or with pre-existing mental and physical health conditions are more likely to report extreme COVID-19-related stressors (Alonzi et al., 2020; Barone et al., 2023; Franic, 2021; Husky et al., 2021; Manchia et al., 2022). Brailovskaia and Margraf (2020) found that pre-pandemic stress among adults predicted COVID-19-related burden at the beginning of the pandemic. Similarly, van Loon et al. (2021) reported that adolescents with specific vulnerabilities including higher stress, maladaptive coping, or internalizing problems before the pandemic experienced higher levels of total and specific COVID-related concerns (getting sick, school, financial problems). In a review of research on the impact of the COVID-19 pandemic on adolescent emotional, social, and academic adjustment, Branje and Morris (2021) indicated that adolescents already at risk before the pandemic experienced increased depressive symptoms, negative affect, loneliness, and lower academic achievement during the pandemic. Kochel et al. (2022) found that college students characterized with a profile of severe psychological symptoms perceived an elevated COVID-19 impact and social maladjustment in comparison to those with mild and moderate symptom profiles.

According to Ghebreyesus, 2020, severe psychosocial stressors conferred by the pandemic are considered to exacerbate mental health problems among those with predisposed susceptibility to psychopathology. Indeed, individuals with prior psychological difficulties or a mental health diagnosis experienced increased disorder and they reported higher levels of COVID-19 related stress (De Luca et al., 2022; Manchia et al., 2022; Murphy et al., 2021; Rettie & Daniels, 2021; Varma et al., 2021). In a review of mental health research during the first year of COVID-19, Aknin et al. (2022) emphasized the need to address continuing psychological distress among individuals with pre-existing psychological conditions and those at potential risk for them. From this perspective prior psychological difficulties would be expected to augment the association between perceived COVID-19 stress and identity distress thereby functioning as a mediator in this relationship.

At the same time Manchia et al. (2022) reported a prolonged impact of COVID-19 on stress-resilience and mental health across the pandemic waves in a reduction of negative affect. However less resilience was seen in general population studies early in the pandemic and there was some evidence of resilience in coping strategies in response to varied challenges.

Several studies indicated positive mental health benefits early in the pandemic to students without a history of psychological difficulties (Haikalis et al., 2022; Meda et al., 2021). Hamza et al. (2021) found an increase in psychological symptoms among students without pre-existing psychological problems relative to those with pre-existing problems in a Canadian sample of university students. The discrepant findings for these groups were attributed to differences in coping strategies. The coping styles of students with prior mental health difficulties were adaptive to restrictions in the pandemic. Conversely, for those without pre-existing psychological problems their regular coping strategies that relied on supportive social interchange were unavailable due to lockdown confinement with ensuing negative impact. In the current study we examined psychological symptoms as a moderator in the relationship between perceived COVID-19 stress and identity distress although there were no predictions because our data was collected during the second wave of the pandemic.

### The Present Study

The present study is from the sixth wave of a repeated cross-sectional project on adaptation at university with students in Spain and Canada. Both countries were in the second phase of the COVID-19 pandemic with severe local restrictions and vaccines unavailable. The timeframe enabled a comparison of students’ functioning before and during the onset of the pandemic. In contrast to earlier research that utilized contrasting contexts depicted by country, this study examined Spanish and Canadian students’ functioning during the COVID-19 pandemic. Given the uncertain nature of this macro-environmental context (Muller et al., 2023), consistency was expected among students in these venues. We predicted: (1) an increase in psychological symptoms and identity distress among students during the pandemic in comparison with pre-pandemic students; (2) positive associations between students’ perceived COVID-19 stress, psychological symptoms, and identity distress; and (3) that psychological symptoms would mediate the linkage between students’ perceived COVID-19 stress and identity distress.

## Method

### Procedure

Data was collected during the 2020/2021 academic year. This was the second wave of COVID-19 before vaccines were available for young adults in both countries. In Canada course delivery was by distance with the campus closed to students; in Spain course delivery alternated once a week between distance delivery and in-class attendance (with physical distancing and wearing masks) to reduce the physical number of students, that is, half of the class in each setting. Students completed online surveys in English and Spanish, respectively. The Spanish version has been used extensively. COVID-19 items were translated into Spanish and back-translated. Instructors announced the research project in their classes. All participants responded to a letter of invitation on their class website that contained a hyperlink to the survey. Respondents completed a survey after providing informed consent by indicating assent. Participants were awarded a bonus point toward their final course grade as a gratuity. This was accomplished by sending IDs of respondents to appropriate instructors for allocation of credit. The study received ethical approval from the Brandon University Research Ethics Committee (BUREC Certificate #200325).

### Participants

Overall, 437 students, 17–29 years of age, completed an online survey including 288 (age_mdn_ = 20 years) in Canada and 149 (age_mdn_ = 18 years) in Spain. Table 1 provides the demographic description of the COVID-19 and the baseline groups.

**Table 1.** Demographic Characteristics of the Canadian and Spanish Students.

Variable	COVID-19		Pre-COVID-19^a^	
	Canada	Spain	Canada	Spain
*N*	288	149	341	107
Females	75.4%	88.4%	80.4%	84.1%
Age	20.8 (2.8)	18.8 (1.6)	19.2 (2.2)	19.8 (1.7)
Self-identified (Race/Culture)				
Caucasian	71.0%	98%	77.8%	100%
Indigenous	8.5%	—	8.2%	—
Asian	11.9%	0.5%	8.6%	—
Black	8.6%	—	5.9%	—
Arab	—	1.5%	—	—
Marital status				
Single	93%	94%	94%	100%
Home community				
Same community	34%	29.5%	30.1%	49.5%
Same province	42.8%	57%	54%	34.6%
Another province	12.6%	12.8%	11.2%	10.2%
Another country	10.5%	7%	4.7%	5.6%
Living situation				
With parents	59%	64.4%	36.4%	78.3%
Spouse/common-law partner	8%	2%	5.3%	—
With roommates (house, apt)	25.7%	28.9%	36.1%	—
Residence/dorm	2.4%	1.3%	11.7%	6.6%
Alone	3.5%	2%	7.6%	7.6%
Other	1.9%	0.7%	2.9%	7.6%
Mother’s education attained				
Median level	University degree or diploma	High school	University degree or diploma	High school
Father’s education attained				
Median level	Some university or college	Some secondary/high school	University degree or diploma	High school

^a^Pre-COVID-19 data are from 2018 (Gfellner & Cordoba, 2020a).

### Measures

The Identity Distress Scale (IDS; Berman et al., 2004) based on the DSM-IV categorization of identity problem was used to assess difficulties with identity development. The IDS measures the extent to which the respondent is experiencing difficulties in each of seven broadly defined identity domains: future planning, career choice, friendships, sexual orientation, religion, values/beliefs, and group loyalties. Items are rated on a 5-point scale from “not at all” (1) to “very severely” (5) to indicate the extent to which the individual has been recently upset, worried, or distressed over each of these identity-related issues. Two additional items assess the extent to which the respondent feels distressed with these issues and the extent to which these issues cause impairment or uncertainty. A final item asks how much time these issues have been a concern. Items are summed for a continuous measure of identity distress. Alpha coefficients for the IDS were .76 and .81 for students in Spain and Canada, respectively.

Psychological symptoms were measured by the Counseling Center Assessment of Psychological Symptoms (CCAPS; Locke et al., 2012) developed to assess clinical disorder and progress in treatment among college students. The 34-item short form (CCAPS-34) includes scales for: depression, general anxiety, social anxiety, eating concerns, hostility, alcohol use, and academic disorder. Items are rated on a 5-point scale from 0 (“not at all like me”) to 4 (“extremely like me”) in terms of frequency during the past 2 weeks. Scale scores are computed as well as a CCAPS Distress Index (CCAPS-DI) that provides a composite score of 20 psychological symptoms and respondents may be categorized in terms of none, moderate, or severe clinical significance (Youn et al., 2015). A recent psychometric analysis is available from Sherman et al. (2021). This study used the CCAPS-DI. Alpha coefficients were .92 and .92 for Spanish and Canadian students, respectively.

Perceived COVID-19 stress was indexed by a single item that asked respondents to rate on a 5-point scale from 1 (“rarely”) to 5 (“much of the time”) the extent to which they have been feeling nervous, anxious, on edge, or stressed because of COVID-19. The use of an abbreviated indicator is considered appropriate when dealing with time-constrained data collection (Chen et al., 2022). Besides, our findings with this single-item measure of perceived COVID-19 stress are consistent with the research literature (Kujawa et al., 2020). As well, it correlated moderately with a 10-item index of global stress over the past 2 weeks, r = .42, *p* < .0002, for Spanish and r = .31, *p* < .0001, for Canadian students.

The COVID-19 stress item is similar to the IDS items as both require global ratings of unique broadly defined indices that refer to distinct domains. Conversely, the CCAPS-24 assesses specific psychological symptoms.

### Data Analysis

**Figure 1.**
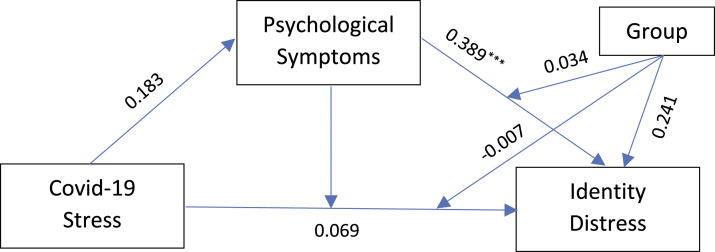
Conceptual model of mediation and moderation with COVID-19 stress (X) the antecedent of identity distress (Y) and psychological symptoms the mediator (M) and group the moderator (W).

The repeated cross-sectional project provided comparative pre-COVID-19 data on the IDS and CCAPS-34 from 2018 for the Spanish and Canadian cohorts (Gfellner & Cordoba, 2020a). T-tests were run with mean scores between the two indices for each group, respectively. Descriptive statistics for all variables in the current study included between-group t-tests and correlations by group. Given the age difference between the groups, age was controlled in the subsequent analyses. The analysis used Hayes (2018) PROCESS macro version 3 model 59 for SAS with decisions about significant effects made with the use of bootstrap confidence intervals. This macro assesses direct and indirect effects in mediated, moderated, and moderated mediation models. Figure 1 diagrams the conceptual model for the second stage moderation of the effects of COVID-19 stress (X) on identity distress (Y) with psychological problems (M) as the mediator and country/group the moderator (W). Moderated mediation was examined given earlier findings of differences in the trajectories for maladaptive exploration in the identity distress linkage with adjustment at university (Gfellner & Cordoba, 2020a).

## Results

The within-group comparisons for pre-COVID-19 with current scores are shown in Table 2. As expected, Canadian students had elevated pandemic psychological symptom scores, Ms: 1.73 (0.87); 1.3 (0.77), t = −6.50, *p* < 0.0001, and identity distress scores did not differ significantly, Ms: 2.29 (0.64); 2.32 (0.72), t = −0.51, *p* < 0.61. Spanish students showed an increase in psychological symptoms during the pandemic, Ms: 1.47 (0.79); 1.2 (0.65), t = −2.901, *p* < 0.004, and a decrease for identity distress scores, Ms: 2.5 (0.57); 2.76 (0.74), t = 2.67, *p* < 0.008.

**Table 2.** Means (SDs), Ranges, and Bivariate Correlations for Study Variables by Group^a^ and Context.

Variable	Identity Distress	Psychological Symptoms	COVID-19 Stress	COVID-19 Mean (SD)	Range	Pre-COVID Mean (SD)^b^
Identity distress	—	.55^θ^	.26^θ^	2.32 (0.72)	1.0–4.44	2.29 (0.64)
Psychological symptoms	.58^θ^	—	.30^θ^	1.73 (0.87)**	0–3.75	1.3 (0.77)**
COVID-19 stress	.30***	.41^θ^	—	2.33 (1.17)	1.0–5.0	—
COVID-19						
Mean (SD)	2.51 (0.67)**	1.47 (0.79)	3.01 (1.23)^θ^	—	—	—
Range	1.11–4.56	0.1–3.9	1.0–5.0	—	—	—
Pre-COVID Mean (SD)	2.76 (0.74)**	1.2 (0.65)**	—	—	—	—

^θ^*p* < .0001, ***p < .001, **p < .01.

^a^Correlations and means for Canadian students are above the diagonal and those for Spanish students are below the diagonal.

^b^Pre-COVID-19 scores are from 2018 (Gfellner & Cordoba, 2020a).

The between-group comparisons for the pre-COVID-19 cohorts indicated significantly higher identity distress scores for Spanish students, M = 2.76 (0.74), than Canadian students, 2.29 (0.64), t = −6.38, *p* < .0001. However, the groups did not differ significantly on psychological symptoms scores, Ms: 1.2 (0.65); 1.3 (0.77), t = 1.21, *p* < 0.23.

As seen in Table 2, during COVID-19 students in Spain had significantly higher scores for identity distress, Ms: 2.51 (0.67); 2.32 (0.72), t = 2.68, *p* < 0.008, and perceived COVID-19 stress, Ms: 3.01 (1.23); 2.33 (1.17), t = 5.66, *p* < 0.0001, but lower scores for psychological symptoms, Ms: 1.47 (0.79); 1.73 (0.87), t = −3.05, *p* < 0.002, than Canadian students. Significant correlations were found between each of the variables for both groups. Identity distress correlated with psychological symptoms and perceived COVID-19 stress, psychological symptoms correlated with perceived COVID-19 stress and with identity distress, and identity distress correlated with perceived COVID-19 stress. These results support the predicted relationships across the study variables.

The results for the moderated mediation model (Figure 1) are given in Table 3. The conditional direct effect of perceived COVID-19 stress (X) on identity distress (Y) was not significant for either students in Spain, Beta = 0.054, SE = 0.044, t = 1.22, *p* < 0.23, CI: [−0.333 to 0.141], or for those in Canada, Beta = 0.061, SE = 0.032, t = 1.90, *p* < .06, CI: [−0.002 to 0.124]. This showed that for both groups perceived COVID-19 stress did not relate significantly to identity distress. As predicted, the conditional indirect effect of psychological symptoms on the association between students’ perceived COVID-19 stress and identity distress attained significance for both Spanish, Beta = 0.1186, BootSE = 0.0310, BootLLCI: 0.0619–0.1819, and Canadian students, Beta = 0.936, BootSE = 0.0217, BootLLCI: 0.0532–0.1402. As predicted, these findings supported psychological symptoms as a mediator in the perceived COVID-19 stress linkage with identity distress. Moderation mediation, Index = 0.0251, BootSE = 0.0372, BootLLCI: [−0.0444 to 0.0990], that is, group differences in the mediation of psychological symptoms between perceived COVID-19 stress and identity distress was not found.

**Table 3.** Summary of the Predictors and Interactions for Identity Distress in the Conceptual Model.

Outcome	Predictor	β	SE	t	*p*-value	*R* ^2^	95% CI
Identity distress	Perceived COVID-19 stress	0.068	0.078	0.873	.383	.337	−0.86 to 0.222
	Psychological symptoms	0.389	0.112	3.496	.0005		0.170 to −0.609
	Group/country	0.241	0.169	1.424	.155		−0.092 to 0.574
	COVID × group	−0.007	0.055	−0.131	.896		−0.115 to 0.101
	Psych symptoms × group	0.034	0.082	0.418	.676		−0.126 to 0.195
	Age (covariate)	−0.013	0.013	−1.023	.307		−0.037 to 0.012

## Discussion

This study examined psychological symptoms in relation to perceived COVID-19 stress and identity distress among Canadian and Spanish students during the second wave of the pandemic. As expected, the comparisons with pre-COVID cohorts indicated an increase in psychological symptoms for both groups. Alternatively, identity distress scores remained stable for students in Canada and decreased for those in Spain. The decline in identity distress scores among Spanish students may reflect a regression toward the mean as well as an outcome of adapting to the indefinite contextual uncertainty of the pandemic (Muller et al., 2023).

Consistent with pre-pandemic research, Spanish students indicated higher identity distress scores than their Canadian cohort (Gfellner & Cordoba, 2017; 2020b; Gfellner et al., 2019) as well as greater perceived COVID-19 stress. Conversely, Canadian students demonstrated elevated psychological symptoms. For both groups, the predicted associations were supported between perceived COVID-19 stress, psychological symptoms, and identity distress, respectively. In this study the CCAPS-Distress Index (CCAPS-DI) mean score for psychological symptoms exceeded pre-pandemic norms for Spanish and Canadian students (Gfellner & Cordoba, 2020a) and met the clinical benchmark for intervention (Youn et al., 2015). Overall, the CCAPS-DI classification breakdown was 21% for severe and 45% for mild clinical symptomology.

These findings are consistent with the pervasive reports of increased mental health problems associated with the pandemic globally among college students (e.g., Araujo et al., 2020; Daniali et al., 2023; Kujawa et al., 2020; Pierce et al., 2020; Planchuelo-Gomez et al., 2020). In a meta-analysis of international population samples Aknin et al. (2022) indicated a gradual return to baseline affective functioning over the first year of the pandemic. These authors reported the greatest incidence of negative affect was among young people, and they underscored the need to focus on psychological distress among individuals with pre-existing diagnosis and those at-risk for psychological problems. Most published studies have considered short-term impacts of the COVID-19 pandemic with population samples, and the reliance on population samples for mental health indicators has been considered to obscure the trajectories of young adults (Daniali et al., 2023). Further research is necessary to examine longer term implications in terms of perceived stressors, health, well-being, psychosocial functioning, and identity development of EA as COVID-19 evolves with changes over time. Fiorillo and Gorwood (2020) emphasized continuing problematic ramifications among college students with the waxing and waning of the pandemic and beyond it. Lokman and Bokting (2022) underscored the role of mental health during and after the COVID-19 pandemic and in future socially challenging times.

Although psychological problems did not moderate students’ perceived COVID-19 stress and identity distress, the findings may reflect a recursive relationship between psychological symptoms and perceived COVID-19 stress with identity distress (Berman et al., 2020). From the clinical perspective Lokman and Bokting (2022) emphasize a bidirectional association between mental health and COVID-19 stress whereby these factors influence one another in relation to functional outcome. Further research is required to investigate individual differences in the psychological functioning of EA as conditions change over the course of the pandemic and in other life-threatening situations. Indeed, changes in the configurations of psychological symptoms and COVID-related stress are being noted as pandemic waves evolve and societal restrictions change (Daly & Robinson, 2022).

Considerable research emphasizes the impact of government restrictions on trajectories of mental health across global populations with young people a high-risk group (Aknin et al., 2022). Barendse et al. (2023) reviewed 12 longitudinal studies from before to the first 6 months of COVID-19 with adolescents in the US, the Netherlands, and Peru. Their findings indicated that the greatest incidence of affective problems was related to the extent of lockdowns mandated in the country. According to Muller et al. (2023) individuals respond to lockdown and distancing in different ways and the perceived stress associated with lockdown and resulting isolation rather than lockdown itself, predicted negative affect. Indeed, the ramifications of lockdown as seen in social isolation and distance education is a substantial predictor of psychological difficulties among university students (Akpinar, 2021; Baltà-Salvador et al., 2021; Buecker & Hortsmann, 2021; Dotson et al., 2022; Halliburton et al., 2021; Hu et al., 2022; Meda et al., 2021; Nowrouzi-Kia et al., 2022).

In the current study Canadian students’ classes were delivered remotely with the university closed. In comparison Spanish students had a hybrid model in which classes alternated weekly on-campus and by distance with half of the students attending each venue on alternate days. Although distancing and wearing masks was enforced, social interaction with peers and instructors was available. Indeed, the opportunity for social engagement is a major resource for EA in dealing with the pandemic (Juvonen et al., 2022). Similarly, Hamza et al. (2021) found that students without prior psychological difficulties demonstrated increased negative affect relative to those with prior reports of psychological symptoms early in the pandemic. These discrepant findings were explained by students in terms of social isolation and the loss of face-to-face communication with peers and instructors as well as academic-related concerns (Ewing et al., 2022). In a longitudinal study of British youth during the second phase of the pandemic Henseke et al. (2022) reported positive effects of less stringent lockdown restrictions including increased social contacts and fewer worries about learning skills and the future. In the present study pre-COVID-19 psychological symptom scores did not differ between Spanish and Canadian students. In comparison for the pandemic cohort, Canadian students evidenced a significantly greater increase in psychological symptom scores relative to the Spanish students. Following Muller et al. (2023), the hybrid educational format in Spain may be reflected in the lower psychological symptoms indicated by these students. Indeed, efforts are being directed toward improving approaches to distance education to alleviate distress and make them amenable to students’ well-being (Baltà-Salvador et al., 2021; Galea et al., 2020; Yeung & Yau, 2022).

As predicted, psychological symptoms mediated the linkage between students’ perceived COVID-19 stress and difficulties with identity development. These results support research that underscores the influence of mental health problems that may exacerbate the impact of critical life events on psychological functioning (Brailovskaia & Margraf, 2020; Branje & Morris, 2021; Husky et al., 2021; Rettie & Daniels, 2021; van Loon et al., 2021). For EA this was evident in difficulties with identity issues, the focal development task of the period. Similarly, Pasupathi et al. (2022) found increases in maladaptive exploration as well as decreases in mental health and academic resilience in a follow-up of first-year university students over the first year of the pandemic, and this was greatest among students with elevated baseline psychological symptoms. It would be useful for further research to address specific identity concerns that are most relevant to students in such precarious times and how these may vary across individuals and contexts (Galliher et al., 2017). Such findings would facilitate more directive approaches to supportive services and intervention (e.g., Meca et al., 2022; Meca et al., 2014).

Several limitations of the study require consideration. The cross-sectional design provides a snap-shot view of students’ functioning early during the pandemic when lockdowns including distance course delivery, curfews, and extreme curtailment of social activities and gatherings began. Longitudinal study is necessary to examine how students adjust to concomitant changes as the pandemic evolves. Nevertheless, the present findings provide benchmark information for subsequent comparison and follow-up. This study is part of a repeated cross-sectional project that focuses on different cohorts of EA from the same contexts. Based on a comparison of repeated cross-sectional with panel studies during the early phase of COVID-19, Zetter et al. (2021) reported some subtle differences as well as advantages of cross-sectional research and the use of both methods allow researchers to draw more convincing conclusions (Aknin et al., 2022). The reliance on self-report data is a limitation; information from parents and other sources such as counseling services would provide a broader perspective of students’ functioning. Unfortunately, such informants are not always readily available, especially in time-sensitive circumstances. The use of qualitative information in a mixed methods design would provide a nuanced view of the issues relevant to students. Finally, the low involvement of males, most notable among Spanish students, is typical in research with college populations (Salkind, 2010). It is a ubiquitous issue that often precludes the examination of sex differences.

As noted elsewhere (Gfellner & Cordoba, 2017; 2020a), our Spanish students represent a selective sample in a professional program of studies with a special resilience in dealing with the contextual stressor of high youth unemployment. Nevertheless, in contrast with prior research the current findings revealed an overarching salience of the COVID-19 pandemic in relation to mental health and identity development of these students in Spain consistent with those in Canada. As predicted, the current results showed that students with psychological difficulties were more vulnerable to the consequences of perceived COVID-19 stress in relation to identity distress. Indeed, even those with mild psychological symptoms may be at risk for problems with identity development. Hence there is a need for focused student resources to address personal adjustment and how to face difficult life events and contextual disruptions. The use of CCAPS-34 is recommended as a viable instrument to document relevant changes in psychological symptoms over time (Youn et al., 2015).

In conclusion, these findings provide some insight into the complexities of adaptive functioning among university students as they negotiate integral life tasks, and they warrant closer consideration of EAs’ resilience in dealing with extreme and unprecedented macro-environmental stressors. The use of coping mechanisms including supportive resources and qualitative information would provide a nuanced perspective of the relevant issues. Further research is essential to address individual as well as cultural differences in the role of such stressors and coping mechanisms in relation to mental health and identity development and how they relate to the functional well-being of EA as the pandemic evolves over time and new crises and adversities emerge.
